# Model-based methods for hospital infection prevention and control: potential and challenges

**DOI:** 10.1186/s44263-025-00229-8

**Published:** 2025-12-05

**Authors:** Jessica R. E. Bridgen, Chris Jewell, Joseph M. Lewis, Stacy Todd, Malcolm G. Semple, Nicholas Feasey, Jonathan M. Read

**Affiliations:** 1 School of Mathematical Sciences, Lancaster University, Lancaster, UK; 2 Liverpool School of Tropical Medicine, Liverpool, UK; 3 University Hospitals of Liverpool Group, Liverpool, UK; 4 Tropical and Infectious Disease Unit, University Hospitals of Liverpool Group, Liverpool, UK; 5 Health Protection Research Unit in Emerging and Zoonotic Infections, Institute of Infection, Veterinary and Ecological Sciences, Faculty of Health and Life Sciences, University of Liverpool, Liverpool, UK; 6 Respiratory Department, Liverpool Institute for Child Health and Wellbeing, Alder Hey Children’s Hospital, Liverpool, UK; 7 School of Medicine, University of St. Andrews, St. Andrews, UK; 8 Centre for Health Informatics, Computing, and Statistics, Lancaster Medical School, Lancaster University, Lancaster, UK

Hospital-acquired infections remain a major challenge for ensuring patient safety. Model-based methods offer a pathway to proactive, data-driven infection control, supporting earlier decision-making and tailored control policies. We outline the potential of these approaches, the key methodological and implementation challenges, and the importance of co-creation for operational use.

## Background

Hospital-acquired infections (HAI) contribute to increased patient morbidity and mortality, and undermine community confidence in healthcare [1]. HAI outbreaks are a major challenge for infection control, requiring targeted control measures to be implemented rapidly. Understanding the role of different transmission pathways is essential for effective infection prevention and control (IPC), yet identifying exactly where and how infections spread within the complex structure of hospital settings is challenging. Modern hospital environments generate vast amounts of data which could be used routinely to identify transmission events and inform control measures, yet this information is underutilised for real-time analytics.

Mathematical modelling offers data-driven approaches to predict and identify transmission hotspots, target interventions, and evaluate control measures in real-time. At a multidisciplinary workshop conducted in 2024 at Lancaster University, clinicians, infection control specialists, and modellers discussed the potential of model-based approaches for infection control, as well as the significant barriers to their adoption; these discussions framed the insights we present here.

Here, we reflect on discussions from the workshop and consider how model-based methods can support the delivery of IPC, identify barriers to implementation, and discuss the importance of interdisciplinary collaboration in translating approaches into practice. This Comment focuses on United Kingdom (UK) health systems; while model-based methods are applicable to other countries, implementation barriers may differ across international contexts.

## Transmission modelling in hospital settings

Mathematical models have been applied to hospital settings to determine key drivers of transmission and to assess IPC efficacy, including improved hand hygiene, patient isolation, and testing strategies [2, 3]. Compartmental transmission models are commonly used to describe disease dynamics at the hospital scale, whereas network models are often employed to integrate individual-level characteristics and staff-patient interactions. Modelling studies have provided critical insights into the transmission of pathogens such as Methicillin-resistant *Staphylococcus aureus*, Vancomycin-resistant *Enterococcus,* and SARS-CoV-2, informing IPC policies on patient cohorting and hygiene [4, 5]. However, these models are often constrained by computational complexity and scalability, either limiting the modelled population to patients and staff within a single ward or simplifying staff-patient-visitor interactions by ignoring temporal or individual-level heterogeneity. Consequently, modelling has predominantly been used to explain outbreaks retrospectively rather than to prospectively predict where interventions should be targeted. This highlights the need to develop methodology that can better balance computational constraints with the ability to handle large complex data and provide actionable insights for IPC practitioners.

## Future potential

Integrated model-based approaches that utilise routinely collected data have great potential to evaluate transmission pathways in real-time, enabling IPC teams to rapidly detect outbreaks, identify transmission hotspots, and implement effective and timely interventions. Mathematical modelling provides a framework for representing hospitals as dynamical systems, incorporating features such as spatial structure, patient and staff movement and interactions, and use of non-ward areas [6]. Modern statistical inference methods can incorporate testing and genomic data with patient and staff location information (e.g. admissions, ward transfers, staff schedules) into models, whilst also accounting for censored data inherent in hospital surveillance. Modelling, therefore, provides an ideal framework to exploit the currently underutilised data routinely collected within hospitals.

For these advances to translate into practice, integration of modelling into healthcare settings must account for how IPC policy is shaped. While IPC policies exist at a national level in the UK, patient and staff pathways are largely determined by local infection control specialists and the requirements of a hospital and healthcare trusts. Flexible model-based approaches provide an opportunity to capitalise on investments in healthcare data collection, and develop IPC strategies specific to a particular hospital. These strategies could be tailored to different pathogens and be adaptable in real time to aid resource allocation and account for staffing constraints. Such approaches would not replace clinical expertise but act as a decision-support tool for IPC teams, potentially transforming the practice of hospital infection control. This will require modelling to be integrated directly with hospital information systems and for modellers and IPC teams to work together closely.

## Key challenges

There are significant barriers to using model-based methods to support real-time decision-making in hospital infection control. These challenges can be categorised into four areas: epidemiology, data, methodology, and implementation.

Uncertainty in the epidemiology of pathogen transmission leads to uncertainty in both parameterisation and model structure. A significant challenge in understanding the epidemiology of hospital outbreaks is the difficulty of distinguishing between a HAI and a community-acquired infection; this becomes more challenging when key parameters are uncertain or highly variable, such as an estimate of community prevalence for the pathogen, prodrome duration, and the proportion of asymptomatic infectious cases. Nonetheless, this classification is crucial to disentangle drivers of transmission during an outbreak. Understanding the relationship between asymptomatic colonisation and symptomatic infection is also important as many bacteria associated with HAI and antimicrobial resistance are typically of low virulence and often present little threat to patients. Their spread through a healthcare facility can pass unnoticed until they cause symptomatic disease in a vulnerable individual. However, it may be this asymptomatic transmission that IPC needs to interrupt. Another challenge is understanding the pathogenesis and natural history of the disease, particularly for novel pathogens. IPC teams require an understanding of complex transmission dynamics, including uncertainty in the primary modes of transmission.

The availability and quality of data also represent major constraints for modelling. Hospital surveillance data are often censored. For instance, while the date a patient tests positive for an infection may be recorded, the date of their infection is unknown. Existing statistical methodology makes incorporating censored surveillance data feasible [7]. Other significant challenges include heterogeneous hospital systems, variability in data collection and storage, and difficulty in accessing clinical information. In England, the National Health Service has made significant investments in subnational secure data environments to improve data access, linkage, and security [8]. However, these environments present their own challenges for researchers and public health specialists, including monetisation of clinical datasets, lack of standardisation across environments, and limited capacity for high-performance computing within environments.

There are numerous methodological challenges in developing a modelling system for infection control in hospitals. One challenge is creating sufficiently flexible transmission models that can incorporate a wide variety of data types, which differ by hospital, pathogen, and mode of transmission. To effectively manage a large outbreak, the entire hospital environment should be considered, requiring the development of hospital-wide network models describing interactions between staff, patients, visitors, and the hospital environment. Existing approaches need further development for efficient scaling and integration with statistical inference or machine learning algorithms. The computational cost of such methods also needs to be evaluated, both in terms of sustainability and run-time, ensuring models can be deployed quickly and updated as outbreaks evolve. This will necessitate the creation of specialised software toolkits that are agile and easily accessible to IPC teams.

Finally, implementing a model-based system for infection control within existing healthcare systems presents its own challenges. Researchers working on these approaches should consider the interpretability and actionability of their analytic outputs, highlighting the importance of co-creation with IPC teams. Integrating a model-based system with a user interface would assist with this, enabling IPC practitioners to engage with relevant parts of the model and visualize key findings. A broader challenge will be building trust in model outputs among health professionals, patients, and the public. This will require transparent model development, ensuring open-source model code, with clear communication of uncertainties, and training sessions with public health specialists. Ethical considerations surrounding patient privacy and data governance should also be addressed. Evaluation through a health technology assessment would help to ensure any such system was safe, equitable and effective. Overcoming these barriers is non-trivial but crucial if model-based methods are to be used reliably and effectively in real-time infection control.

## Research policy interface

IPC policy is made under considerable situational uncertainty, whereby the apparent infection landscape differs from the true infection landscape. Evidence from the UK COVID-19 Inquiry shows that in pandemic circumstances, even when HAI was recognised promptly through robust surveillance, commissioners and regulators struggled to address the root causes of poor IPC in severely affected hospitals. Moreover, they were unable to learn from and share the lessons of the very best IPC from hospitals that successfully suppressed HAI despite high activity [9]. Model-based methods can help to address this by integrating multiple data sources to provide evidence-based estimates of the true state of infection, beyond what can be inferred by surveillance data alone.

## Conclusions

Advancing the use of model-based methods for hospital IPC will require a concerted, collaborative effort to tackle the challenges outlined above. Co-creation between infection control specialists, clinicians and modellers will ensure that analytic outputs are easily interpretable and actionable, while remaining policy-relevant. Furthermore, transparency in model structure and communication of uncertainty will be vital in helping to build trust among decision-makers and ensure that these approaches can support current efforts. Model-based methods offer a pathway to proactive, data-driven IPC, supporting timely decisions and tailored, effective control strategies. Given that the necessary data and clinical demand for data-driven insights already exist, the potential of model-based methods for hospital infection control now depends on cross-disciplinary collaboration, strategic funding, and methodological development.

## Data Availability

No datasets were generated or analysed during the current study.
